# Esophagus Segmentation from 3D CT Data Using Skeleton Prior-Based Graph Cut

**DOI:** 10.1155/2013/547897

**Published:** 2013-08-29

**Authors:** Damien Grosgeorge, Caroline Petitjean, Bernard Dubray, Su Ruan

**Affiliations:** ^1^Université de Rouen, LITIS EA 4108, 22 Boulevard Gambetta, 76183 Rouen Cedex, France; ^2^Centre Henri Becquerel, rue d'Amiens, 76038 Rouen Cedex 1, France

## Abstract

The segmentation of organs at risk in CT volumes is a prerequisite for radiotherapy treatment planning. In this paper, we focus on esophagus segmentation, a challenging application since the wall of the esophagus, made of muscle tissue, has very low contrast in CT images. We propose in this paper an original method to segment in thoracic CT scans the 3D esophagus using a skeleton-shape model to guide the segmentation. Our method is composed of two steps: a 3D segmentation by graph cut with skeleton prior, followed by a 2D propagation. Our method yields encouraging results over 6 patients.

## 1. Introduction

**Figure 1:**
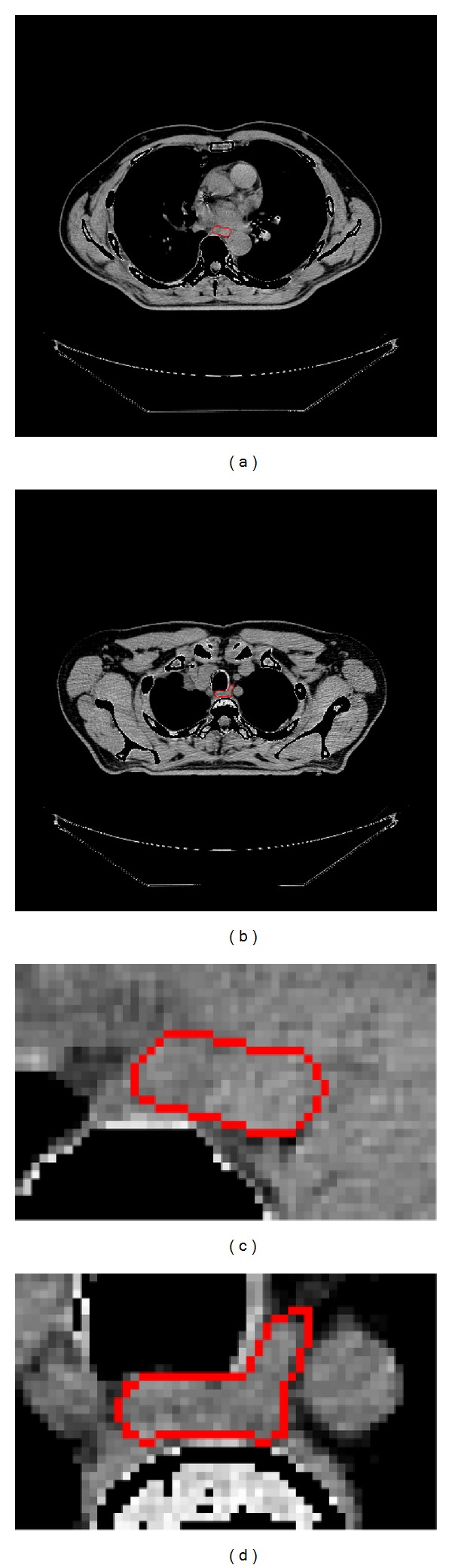
CT scan ((a), (b)) and cropped ROI from CT scan ((c), (d)) with esophagus manually segmented in red. Note the variable shape of the esophagus and how its grey levels are similar to surrounding tissues.

 Lymphoma is a common tumor in the mediastinum, which is often located close to the trachea and the esophagus. Before radiotherapy, organs at risk such as heart, lung, and esophagus. must be outlined, in order to minimize the quantity of irradiation. In particular, the esophagus is very challenging to delineate because of the really low contrast of its boundaries, its complex shape and its inhomogeneous appearance (Figure 1). Today, the segmentation of the esophagus is performed manually by clinicians and is a tedious task, prone to intra- and interobserver variabilities.

Due to its difficulty, the literature on (semi)automatic esophagus segmentation is quite light. In [1], Rousson et al. proposed a two-step segmentation method in cardiac CT: first, the esophagus centerline is extracted using probabilistic spatial and appearance modeling, and then the outer surface of the esophagus is extracted using multiple and coupled ellipse fittings. However, the method requires as input two points on the centerline of the esophagus and manual segmentation of the aorta and the left atrium. In [2], Ragan et al. segment several organs using deformable models but fail to accurately contour the esophagus. In [3], Huang et al. propose a semiautomated method consisting in manually drawing one contour in an axial slice, which is propagated to other slices of based registration, but no quantitative evaluation is proposed in this paper. Fieselmann et al. in [4] propose, from several contours in axial slices provided by user input, to interpolate all missing contours in the frequency domain. A fully automatic method is presented in [5] by Feulner et al. that consists in first finding the approximate shape using a “detect and connect” approach, and then a classifier is trained to find short segments of the esophagus which are approximated by an elliptical model.

Most of these methods require a significant amount of user input or are based on an elliptical model. As shown in Figure 1, the esophagus is a deformable organ and has a more complex shape which can hardly be approximated by an elliptic shape. In this work we propose a method to segment the esophagus on thoracic CT scans in two steps: (i) a 3D segmentation by graph cut using a skeleton prior and (ii) a detection of the first slice of inaccurate segmentation, an oversegmentation due to the same gray level of neighboring organs such as aorta, and a 2D segmentation by graph cut for the next slices. Our method requires a very simple user input, has a low computational cost, and gives encouraging preliminary results on 6 patients. 

## 2. Method: 3D Graph Cut Segmentation with Shape Prior

 In this section, we first outline the graph cut segmentation framework as described in [6]. Then, we introduce our method, which includes the construction of the skeleton model, the 3D segmentation using graph cuts, and the 2D graph cut propagation.

### 2.1. 3D Graph Cut Segmentation

 Let us consider the volume *V* as a graph *𝒢* = 〈*𝒱*, *ℰ*〉, where *𝒱* is the set of nodes (voxels) and *ℰ* the set of edges. Each pair of nodes (*p*, *q*) ∈ *ℰ* in a neighborhood *𝒩* is connected by a segment called *n-link* and weighted by *B*_*p*,*q*_, a regularization or boundary term, designed to provide spatial coherence in a neighborhood of voxels. *B*_*p*,*q*_ is typically defined as

(1) $$\begin{array}{c} B_{p,q}\propto \exp\left(-\frac{{\left(V_{p}-V_{q}\right)}^{2}}{2\sigma^{2}}\right)\cdot \frac{1}{\mathrm{dist}\left(p,q\right)}, \end{array}$$

where *V*_*p*_ and *V*_*q*_ are the gray levels of voxels *p* and *q*, dist⁡(*p*, *q*) the Euclidean distance between *p* and *q*, and *σ* a constant usually related to acquisition noise. Consider two additional nodes, called terminal nodes: the source *𝒮* representing the object *𝒪* (in our case, the RV cavity) and the sink *𝒯* representing the background *ℬ*. Each node *p* ∈ *𝒱* is connected to the terminal nodes *𝒮* and *𝒯* by two respective segments called *t-links* and weighted by the so-called region term denoted by *R*_*p*_ and defined by

(2) $$\begin{array}{c} R_{p}\left(\omega \right)=-\text{lnPr}\left(V_{p}\;|\;\omega \right), \end{array}$$

where Pr(*V*_*p*_ | *ω*) is the likelihood of observing *V*_*p*_ given that voxel *p* belongs to class *ω* that is intensity distribution of class *ω*.

A cut *𝒞* in the graph consists in cutting *t-links* and *n-links* to attribute a label *𝒪* or *ℬ* to each voxel *p* of the image, which boils down to segment the volume. The energy of a cut *𝒞* is defined by

(3) $$\begin{array}{c} E\left(𝒞\right)=\sum_{p\in 𝒱}^{}R_{p}\left(\omega_{p}\right)+\lambda \sum_{p,q\in 𝒩}^{}B_{p,q}\cdot \delta \left(\omega_{p}\neq \omega_{q}\right), \end{array}$$

where *δ*(*ω*_*p*_ ≠ *ω*_*q*_) is 0 if *p* and *q* have the same label, 1 otherwise. The 3D optimal segmentation is obtained by searching for the cut of minimal energy. This global search can be very efficiently performed due to mincut-maxflow algorithms, in polynomial time [7].

### 2.2. Proposed Graph Cut Segmentation Framework Using Shape Prior

 We propose a two-step method to segment the esophagus on thoracic CT scans based on graph cuts with a skeleton prior. In Section 2.2.1, we present the construction of a skeleton model based on a principal component analysis (PCA). Using this model, the first step of the method consists in segmenting the esophagus based on 3D graph cut (Section 2.2.2). We show 3D segmentation overestimate from a certain slice, taking aorta into esophagus segmentation. In this case, the variability of the skeletons is so important that the 3D shape model cannot well guide the segmentation. The second step consists then in detecting this slice, called breaking slice *bs* in the following, and realizes from it 2D segmentation by graph cut with a skeleton prior for the rest slices (Section 2.2.3).

#### 2.2.1. Construction of the Skeleton Model

**Figure 2:**
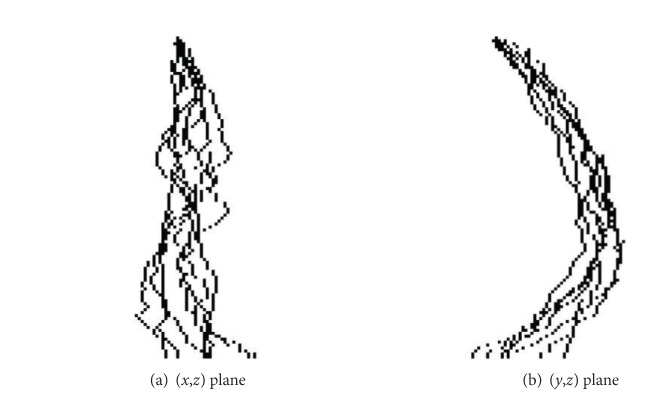
Superimposition of skeletons after alignment.

 Let us consider *N* 3D esophagus obtained by expert's manual delineation on CT scans. On each slice *s*, a gravity center *c*_*s*_^*n*^ can be computed 1 ≤ *n* ≤ *N* and a skeleton is made of all gravity centers *C* ^*n*^ = {*C*_*i*_^*n*^, *C*_2_^*n*^,…, *C*_*S*_^*n*^} to where *S* is the number of slices. Gravity centers are first decimated to allow skeletons to have the same number of points. Following the definition of a point distribution model (PDM) as first introduced in [8], skeletons are then rigidly aligned using a Procrustes analysis (see Figure 2). A mean skeleton $\overset{-}{\Phi }$ is then computed:

(4) $$\begin{array}{c} \overset{-}{\Phi }=\frac{1}{N}\sum_{n=1}^{N}C^{n}. \end{array}$$

A PCA is then performed on the set of centered skeletons and yields eigenmodes denoted by Φ_*i*_, with *i* = 1,…, *N*, and their associated eigenvalues, denoted by *λ*_*i*_. The number *k* ≤ *N* of eigenmodes is retained, with *k* chosen large enough to account for the most important skeleton variations present in the training set.

Let us now describe how a single skeleton prior is computed from the PCA. Our aim is to isolate areas of variation of the mean skeleton, for each principal axis. We thus generate deformed skeleton instances for each axis:

(5) $$\begin{array}{c} \gamma_{i}^{\alpha }=\overset{-}{\Phi }\pm \alpha \sqrt{\lambda_{i}}\Phi_{i},\;\forall i=1,\ldots ,k,\;\;\alpha \in \left[-3:3\right]. \end{array}$$

*α* has to be bounded in order to represent variations in the training set of 3D esophagus. Classical boundary values of *α* in PCA framework are [−3; +3] [9]. A lower value would not capture all variations given by the training set. A higher value would include too large variations and would not ensure smooth deformations. All possible positions of skeletons are summed up in *γ*_*i*_^*α*^. A prior volume is then obtained by computing the minimum distance between a point *p* and its nearest skeleton point:

(6) $$\begin{array}{c} P_{3\text{D}}\left(p\right)=\underset{\alpha ,i}{\min}\mathrm{dist}\left(p,\gamma_{i}^{\alpha }\right), \end{array}$$

where dist⁡(*p*, *γ*_*i*_^*α*^) is the euclidean distance between a point *p* and its nearest gravity center from all possible skeletons. Thus, the lower the distance, the higher the probability to be inside the esophagus.

#### 2.2.2. 3D Segmentation Using Graph Cuts

 Now how can this prior map be integrated into the graph cut framework? In the literature, additional energy terms on the *t-links* [10] or the *n-links* [11] are added to the graph cost function. In any case, the prior must be rigidly registered onto the image to be segmented. The user is thus required to point out two landmarks: inside the esophagus, in the first and last slices of the volume. Esophagus intensity values are in the range −100–200 in CT scans. The prior map *P*_3D_(*p*) gives the distance of pixel *p* to a possible skeleton of the esophagus. We thus suggest that the shape prior contributes to weighting *t-links*. The region term with skeleton prior *R*_*p*_^*S*^ can straightforwardly be defined with

(7) $$\begin{array}{c} R_{p}^{S}\left(𝒪\right)=\{\begin{array}{cc} +\infty & \text{if}\;\;V_{p}<-100\;\;\text{or}\;\;V_{p}>200\\ \frac{{\left(P_{3\text{D}}\left(p\right)\right)}^{2}}{2\sigma_{r}^{2}} & \text{otherwise}, \end{array}\\ R_{p}^{S}\left(ℬ\right)=\{\begin{array}{cc} 0 & \text{if}\;\;V_{p}<-100\;\;\text{or}\;\;V_{p}>200\\ \frac{{\left(P_{3\text{D}}\left(p\right)\right)}^{2}}{2\sigma_{r}^{2}} & \text{otherwise} \end{array} \end{array}$$

with *V*_*p*_ being the gray level of voxel *p*, *P*_3D_(*p*) the distance prior map, and *σ*_*r*_ a parameter defining what value of distance can be considered as being near to the possible skeleton.

We use the classical definition of *B*_*p*,*q*_ to weight the *n-links* as defined in (1). The final energy of a cut *𝒞* for our 3D graph integrating a shape prior is then

(8) $$\begin{array}{c} E\left(𝒞\right)=\lambda \sum_{p,q\in 𝒩}^{}B_{p,q}\cdot \delta \left(\omega_{p}\neq \omega_{q}\right)+\sum_{p\in V}^{}R_{p}^{S}\left(\omega_{p}\right), \end{array}$$

where *λ* weights the relative contributions of the *n-link* and *t-link* terms.

**Figure 3:**
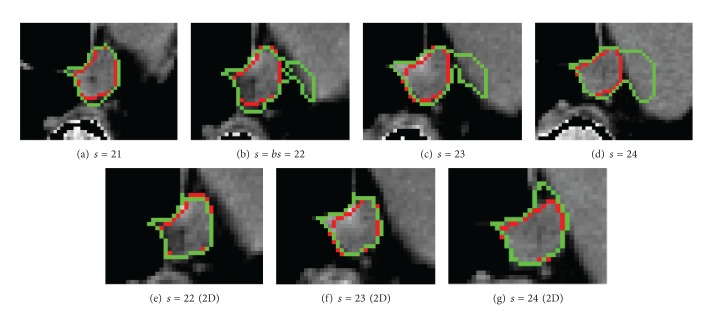
Example of the breaking slice detection (at slice *s* = 22). *s* is the slice number. (a)–(d) Segmentation with 3D graph and (e)–(g) results of the 2D segmentation. Red: manual contour; green: automatic contour.

However, as esophagus is a moving cavity, compressed by the other organs, possible skeletons are various. Moreover, esophagus shares the same gray level with neighboring organs (in particular the aorta). As a result, from a certain slice, an oversegmentation is observed with the 3D graph (see Figure 3(c)). We propose to detect slices with oversegmentation and use 2D propagation by graph cut to improve segmentations.

#### 2.2.3. 2D Propagation by Graph Cut

 To detect the slice position where the oversegmentation begins, we use a simple heuristic: esophagus area does not change consequently from one slice to the other. Our aim is to detect the slice where oversegmentation starts by browsing through slices from top to bottom. This slice level is called breaking slice *bs* and is determined by

(9) $$\begin{array}{c} bs=\underset{s\in S}{\min}:\frac{{𝒜}_{s}}{{\overset{-}{𝒜}}_{1,\ldots ,s-1}}<1-\ell \;\text{or}\;1+\ell <\frac{{𝒜}_{s}}{{\overset{-}{𝒜}}_{1,\ldots ,s-1}}, \end{array}$$

where *𝒜*_*s*_ is the esophagus area of slice *s*, ${\overset{-}{𝒜}}_{1,\ldots ,s-1}$ the mean esophagus area of previous slices, and *ℓ* the threshold of area variation. From *bs*, we use a 2D segmentation by graph cut with prior for each slice. The final energy of the 2D graph is the same as 3D graph, as defined in (8). The prior is defined by the distance to the gravity center of the previously segmented slice *s* − 1:

(10) $$\begin{array}{c} P_{2{\text{D}}_{s}}\left(p\right)=\mathrm{dist}\left(p,c_{s-1}\right), \end{array}$$

where *c*_*s*−1_ is the gravity center of the previous segmented slice *s* − 1.

## 3. Experimental Results and Discussion

 The proposed segmentation method has been applied on thoracic CT scans of 6 patients (which have been acquired with different scanners). Each CT volume includes between 73 and 108 slices. Voxel size is 0.98 × 0.98 × 2.0 mm or 0.98 × 0.98 × 2.5 mm.

### 3.1. Skeleton Model Construction and Method Parameterization

 Following a leave-one-out cross-validation strategy (LOOCV), 6 skeleton models are constructed using a training set of 5 esophagus skeletons. Preliminary registration is performed by superposing the first and the last gravity centers of each skeleton on an arbitrary reference.

Parameters are derived empirically: *σ*_*r*_ = 7, *σ* = 40, *ℓ* = 0.5, and *λ* = 2 for 3D and 2D graph segmentations. The implementation of Boykov and Kolmogorov of the mincut-maxflow algorithm (available online at http://pub.ist.ac.at/~vnk/software.html) is used to compute the cut of minimal cost in the graph [7].

### 3.2. Segmentation Results

 Our segmentation algorithm is run on 6 patients following an LOOCV strategy. For each volume, the user is required to point out two landmarks in order to register the skeleton prior: a point inside the esophagus in the first slice and a point inside the esophagus in the last slice. Our segmentation results are compared to manual ground truth through the Dice metric (DM), a standard overlap measure for comparing two surfaces. The Dice coefficient is given for 3D results and 2D results after slice *bs* and combined 3D and 2D results. The Dice score is stopped from being computed when a zero value is found and considered next slices as not segmented. Proportions *P* of segmented slices considering *bs* over the total number of slices of esophagus are also computed. Results are provided in Table 1.

**Figure 4:**
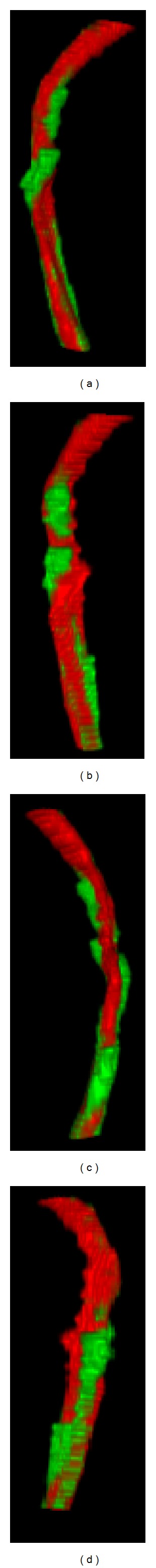
Different views of 3D reconstruction of the esophagus. Red: manual contour; green: automatic contour.

 Not surprisingly, as shown in Table 1, segmentation results are better for the first slices of esophagus with 3D segmentation, as the esophagus contours are better defined.  Figure 3 shows the breaking slice *bs* detection and the segmentation differences between the 3D and 2D methods. An example of 3D reconstruction of an esophagus is also shown in Figure 4.

The use of 2D segmentation allows avoiding overestimation given by 3D graph method in slices much more difficult to segment and to obtain better results. Considering this challenging application, our method yields encouraging results. However, as shown in Table 1, an average of 88.9% (±11.9%) of esophagus slices are segmented with our method (from top to bottom). Room for improvement is left in the last slices of esophagus. Note that, thanks to the well-known high computational efficiency of graph cut, the computation time of our method is about 15 seconds by patient on a regular PC hardware, a time compatible with clinical practice.

## 4. Conclusion and Perspectives

 In this paper, we have presented a method to segment the esophagus in CT scanner, based on a graph cut approach with incorporation of a shape prior. The shape model is a 1D PDM, constructed via a PCA from a set of skeletons of the esophagus obtained by manual segmentation. This shape model is then integrated into the graph cut cost function as prior term, in order to guide the segmentation. A combined 3D and 2D segmentation method is proposed. Results have been presented over 6 3D CT scans. If results are satisfying for the first slices, room for improvement is left in the remaining ones.

Apart from a validation on a larger database of patients, future works will focus on improving the 3D model of skeleton. We are currently investigating how to use air hole to guide the 2D segmentation process. Other works on 3D curve shape models such as [12] and medial tubular models [13] could also be fruitfully investigated.

## Figures and Tables

**Table 1:** Mean (±standard deviation) Dice metric (DM) between automatic and manual delineations of the esophagus and proportion *P* of segmented slice considering the total number of slices.

	DM	*P* (%)
3D Seg	0.78 ± 0.03	33.5 ± 5.1
2D Seg	0.48 ± 0.09	55.4 ± 13.8

3D + 2D Seg (total)	0.61 ± 0.06	88.9 ± 11.9
